# Current Evidence, Selective Indications, and the Role of Lymph-Node Assessment in Intraoperative Frozen Section in Thyroid Cancer Surgery: A Literature Review

**DOI:** 10.3390/jcm15041611

**Published:** 2026-02-19

**Authors:** Gregorio Scerrino, Marco Marcianò, Bianca Vicari, Maria Aurora Bullaro, Renato Di Vuolo, Pierina Richiusa, Giuseppina Orlando, Vito Rodolico, Giuseppina Melfa

**Affiliations:** 1 Unit of Endocrine Surgery, Department of Surgical Oncological and Oral Sciences, Policlinico “P. Giaccone”, University of Palermo, Via Liborio Giuffré 5, 90127 Palermo, Italy; gregorio.scerrino@tiscali.it; 2 Unit of General and Emergency Surgery, Department of Surgical Oncological and Oral Sciences, Policlinico “P. Giaccone”, University of Palermo, Via Liborio Giuffré 5, 90127 Palermo, Italy; mm.94@hotmail.it (M.M.); bianca.vicari@community.unipa.it (B.V.); aurorabullaro1812@gmail.com (M.A.B.); renato.divuolo@community.unipa.it (R.D.V.); irene_melfa@yahoo.it (G.M.); 3 Department of Health Promotion Sciences Maternal and Infantile Care, Internal Medicine and Medical Specialties (PROMISE), University of Palermo, 90127 Palermo, Italy; pierina.richiusa@policlinico.pa.it (P.R.); vito.rodolico@policlinico.pa.it (V.R.)

**Keywords:** frozen section, thyroidectomy, papillary thyroid carcinoma, central neck dissection, medullary thyroid carcinoma, desmoplastic stromal reaction

## Abstract

**Background/Objective**: Intraoperative frozen section (FS) has long been used in thyroid surgery; however, its routine usefulness has shrunk with high-resolution ultrasound, standardized cytology, and molecular diagnostics. This narrative review synthesizes >20 years of evidence to clarify where FS still adds clinically meaningful value and where it does not. **Methods**: This study constitutes a narrative review of the contemporary literature spanning more than two decades, integrating prospective and retrospective evidence on FS performance in indeterminate/suspicious cytology (Bethesda III–V), NIFTP recognition, central compartment lymph nodes in papillary thyroid carcinoma (PTC), and prognostic intraoperative markers in medullary thyroid carcinoma (MTC). It also examines how guidelines and emerging technologies influence intraoperative decision-making. **Results**: FS shows high specificity but limited sensitivity in Bethesda III–IV and Bethesda V cytology, offering minimal incremental diagnostic help in the settings with greatest preoperative uncertainty. FS cannot diagnose NIFTP because definitive classification requires complete capsular examination, incompatible with intraoperative pathology workflows. The most consistent value is FS of central compartment lymph nodes in PTC: it reliably detects macrometastases, enables real-time tailoring of surgical extent, and may reduce staged completion operations. In MTC, intraoperative assessment of desmoplastic stromal reaction appears promising as a prognostic marker; however, it remains investigational and not yet embedded in standard surgical algorithms. Guidelines internationally therefore de-emphasize routine FS. Meanwhile, evolving tools (quantitative imaging, molecular profiling, AI) are reshaping intraoperative decision-support, increasingly positioning FS as one component of a multimodal framework rather than a standalone arbiter. **Conclusions**: Routine FS is largely unsupported in modern risk-stratified thyroid practice due to the low sensitivity in key cytologic gray zones and inability to diagnose NIFTP. Its selective strength persists most clearly in central neck lymph-node assessment in PTC, where it can directly change intraoperative management. Future operative strategies will likely treat FS as an adjunct—contextualized and amplified by imaging, molecular data, and AI—rather than as a default diagnostic step.

## 1. Introduction

Thyroid nodules are among the most common findings in endocrine practice, with a prevalence exceeding 50% in adults when high-resolution ultrasound is employed [1]. Although most nodules are benign, Fine-needle Aspiration Biopsy (FNAB) identifies malignancy in approximately 9–13% of sampled lesions [2,3]. Over recent decades, improved imaging quality, systematic ultrasound-based risk stratification, and the widespread use of FNAB have markedly refined preoperative assessment and reduced the historical need for intraoperative diagnostic procedures [4,5].

A major milestone in this evolution has been the introduction of the Bethesda System for Reporting Thyroid Cytopathology (BSRTC) in 2009 and its 2017 revision, which standardized FNAB terminology, clarified malignancy risks across six diagnostic categories, and facilitated consistent clinical decision-making [6,7]. Indeterminate or suspected categories (Bethesda III–IV–V) continue to present challenges, particularly follicular-patterned lesions for which cytology cannot distinguish between benign and malignant tumors without demonstration of capsular or vascular invasion [8]. This diagnostic limitation historically supported the use of intraoperative frozen section (FS) as a means to establish malignancy, convert lobectomy to total thyroidectomy when necessary, and avoid second-stage procedures.

However, the utility of FS for thyroid nodules has progressively been questioned. Although FS maintains very high specificity, its sensitivity for follicular-patterned lesions—including follicular neoplasms, follicular carcinoma, follicular-variant papillary thyroid carcinoma, and NIFTP-spectrum entities—is consistently low due to freezing artifacts and inability to assess capsular invasion [9,10,11]. Several studies have demonstrated that FS rarely alters intraoperative management in indeterminate nodules and may introduce additional costs and operative time without a corresponding clinical benefit [10,11,12]. Consequently, professional societies increasingly advise against routine reliance on FS for nodular diagnosis, emphasizing instead a preoperative risk-stratified strategy supported by ultrasound, FNAB, and, where appropriate, molecular testing [5,7].

However, the role of FS has not vanished; rather, it has shifted. In papillary thyroid carcinoma (PTC), cervical lymph-node metastases (especially in the central compartment) are both common and difficult to detect preoperatively due to anatomic constraints and limited ultrasound sensitivity, often reported at 20–31% [13,14,15]. In this setting, FS may provide timely confirmation of metastatic disease and guide selective central neck dissection, potentially avoiding incomplete staging or later reoperation. Emerging evidence suggests that FS performs particularly well in identifying macrometastases, which are most likely to influence recurrence risk and operative planning [14].

Contemporary thyroid cancer management, with its emphasis on risk-adapted surgery, selective use of total thyroidectomy, recognition of the very low-risk follicular thyroid neoplasm so-called “Non-Invasive Follicular Thyroid Neoplasm with Papillary-Like Nuclear Features” (NIFTP), and minimization of avoidable morbidity, demands renewed clarity regarding the specific niches in which FS remains clinically relevant. Modern practice increasingly requires a balanced approach: preoperative diagnostic precision complemented—only when appropriate—by intraoperative tools capable of resolving uncertainties that meaningfully impact surgical strategy.

This review aims to provide an updated, integrated appraisal of intraoperative frozen section in thyroid cancer surgery, examining its declining role in indeterminate thyroid nodules, its emerging utility in lymph-node assessment, and its alignment with contemporary risk-stratified surgical paradigms.

## 2. Material and Methods

This work was designed as a narrative, theme-oriented review aimed at summarizing and critically interpreting contemporary evidence on the intraoperative use of FS in thyroid cancer surgery.

A structured but non-systematic literature search was conducted to identify relevant studies addressing the intraoperative use of frozen section concerning this focus. Searches were performed in PubMed/MEDLINE, Scopus, and Web of Science using predefined keyword combinations related to frozen section, thyroid pathology, cytological categories, lymph-node assessment, and intraoperative decision-making. The search strategy was intentionally broad and theme-oriented, consistent with the narrative design of the review. A structured literature search was performed in PubMed/MEDLINE, Scopus, and Web of Science up to 2025. Search terms included combinations of “frozen section”, “thyroid nodule”, “indeterminate cytology”, “Bethesda”, “papillary thyroid carcinoma”, “central lymph node”, “high-risk histology”, and “intraoperative diagnosis”. Additional articles were identified through manual screening of reference lists from key papers and relevant reviews.

Three independent reviewers (BV, MAB, RDV) screened titles, abstracts, and full texts. Discrepancies were resolved by consensus. Because this was not a systematic review, no predefined inclusion or exclusion criteria were imposed; instead, studies were selected based on their clinical relevance, methodological clarity, and contribution to one of the predefined thematic domains. Eligible sources included original research articles, observational studies, meta-analyses, consensus statements, and international guidelines.

The retrieved literature was subsequently categorized into eight analytical sections reflecting the major clinical domains in which FS has been historically used or is currently debated:

- Reappraisal of Frozen Section: Historical Context and Contemporary Criticisms (5 articles);
- FS in Indeterminate or Suspicious Thyroid Cytology (Bethesda III–IV–V) (6 articles);
- The Diagnostic Challenge of NIFTP: Why Frozen Section Fails to Achieve Definitive Identification (4 articles);
- FS for Central Compartment Lymph Nodes in PTC (10 articles);
- FS for High-Risk Histopathological Features (2 articles);
- FS in Medullary Thyroid Carcinoma (6 articles);
- Current Recommendations from International Guidelines (7 sources);
- Future Directions and Emerging Roles, Including Artificial Intelligence (5 articles).

Data extraction focused on diagnostic performance (sensitivity, specificity, PPV, NPV), surgical impact, limitations, and alignment with current risk-stratified thyroid cancer management.

The synthesis of the retrieved data enabled the identification of areas of consensus and controversy, as well as the recognition of studies that substantially informed the field, resulting in a consolidated and comprehensive interpretation of the literature.

Only studies from which qualitative data were directly extracted and analyzed were included in the methodological assessment and enumerated in the Methods section. Additional articles may be cited within individual subsections to provide contextual background or support specific statements, but were not used for the core qualitative analysis underpinning the aims of this review.

Owing to the heterogeneity of study designs and outcomes, no formal quality scoring, quantitative synthesis or meta-analysis was performed. All findings are presented as a qualitative, integrative synthesis consistent with the goals of a narrative review.

The articles selected for formal qualitative analysis are reported in eight thematic tables, each aligned with a specific subsection and analytical focus of the review.

## 3. Results of the Narrative Evidence Synthesis

In accordance with the thematic framework defined in the Methods section, the findings from the retrieved literature are presented across the seven previously established domains.

Each thematic domain is examined to integrate converging evidence, highlight points of divergence, and outline the specific contributions of relevant studies within the broader context of contemporary thyroid cancer surgery.

### 3.1. Reappraisal of Frozen Section: Historical Context and Contemporary Criticisms

As early as 2005, LiVolsi and Baloch [15] highlighted the fundamental limitations of intraoperative frozen section for thyroid nodules, outlining several principles that remain highly relevant today. First, when FNAB yields a diagnosis of papillary thyroid carcinoma, intraoperative consultation provides no additional diagnostic value, as the false-positive rate of competent cytopathology is exceedingly low. Second, frozen section offers minimal utility in follicular and Hürthle cell neoplasms, since the distinction between benign and malignant disease needs the evidence of capsular and vascular invasion: it cannot be showed on frozen material.

It should also be acknowledged that FS is most informative when FNAB is reported as “suspicious for papillary carcinoma,” as intraoperative cytologic evaluation may clarify nuclear features sufficiently to guide the extent of surgery.

Finally, the authors strongly advised against performing frozen section on incidentally detected subcentimeter nodules: freezing artifacts may compromise permanent histology, and even when malignant, such lesions typically represent clinically insignificant papillary microcarcinomas, rendering any intraoperative escalation in surgical extent both unnecessary and potentially inappropriate.

In a study conducted in the early phase after the BSRTC was implemented, this new terminology significantly reduced ambiguous FNAB reports; however, it seems to have no effect on the accuracy or frequency of frozen sections. FS remains diagnostically limited even in an optimized cytologic environment. Its “crisis” appears not merely as a consequence of outdated reporting systems, but reflects structural limitations that persist despite improved preoperative risk stratification [16].

Recent evidence further indicates that molecular profiling (particularly BRAF-directed analysis) now provides a more accurate biological predictor of malignancy than intraoperative morphology, thereby diminishing the added value of frozen section and shifting surgical decision-making toward genotype-based rather than histology-based algorithms [17,18].

Mallick et al. (2019) [19] showed that, in a cohort managed after the introduction of molecular testing, frozen section altered surgical management appropriately in only 2.1% of cases. In contrast, FS misdirected surgery more frequently, leading to both overtreatment and undertreatment, and offered no additional value in the large subgroup of indeterminate nodules already characterized by molecular assays. These findings reinforce that, in the modern diagnostic era, the intraoperative morphology provided by FS is not only limited but may be clinically misleading, especially when compared with the far greater predictive reliability of molecular profiling.

The key studies addressing the historical role and contemporary criticisms of frozen section are summarized in Table 1.

### 3.2. Frozen Section in Indeterminate or Suspicious Thyroid Cytology

The application of FS has also been examined in the setting of suspicious cytology. Although Abu-Ghanem et al. [20] reported a high specificity for FS in Bethesda V nodules, the negative predictive value of only 50% reinforces the inherent limitations of this technique: FS is dependable when positive but provides little reassurance when negative. Its performance is even poorer in Bethesda III–IV nodules, where FS misclassified 75% of malignancies despite a nominal specificity of 100%, underscoring that the technique is least informative precisely where additional intraoperative guidance would be most valuable [11]. Further highlighting these limitations, Eilsberger et al. [21] showed that FS is often used as a substitute for adequate preoperative assessment, thereby amplifying its diagnostic shortcomings and increasing the risk of misdirecting surgical decision-making.

These observations are consistent with the findings of a large Brazilian series of 2040 intraoperative FS, which reported excellent overall accuracy (98%) but reproduced the same pattern of limitations: false negatives clustered in minimally invasive follicular carcinomas, false positives occurred in hyperplastic and inflammatory lesions, and 11% of cases remained indeterminate [22].

Additional contemporary evidence further reinforces the inherent limitations of frozen section in indeterminate cytology. It has been highlighted that, despite its historical use, FS rarely alters surgical strategy in Bethesda III–IV nodules, where decision-making is now primarily driven by preoperative risk assessment [23]. More recent institutional data confirmed moderate sensitivity for FS across Bethesda III–V lesions, with minimal diagnostic gain even when combined with imprint cytology, underscoring persistent variability and limited intraoperative usefulness [24]. Consistent with these findings, the comprehensive review by Uludag et al. showed that FS performs particularly poorly in follicular-patterned lesions, where evaluation of capsular or vascular invasion is not feasible intraoperatively, and may even compromise final pathology by introducing freezing artifacts [12].

Thus, even in high-volume settings with rigorous pathology workflows, frozen section shows its greatest weaknesses precisely in the follicular-patterned and cytologically indeterminate nodules where guidance is most needed.

The diagnostic performance of frozen section in indeterminate or suspicious cytology is summarized in Table 2.

### 3.3. Diagnostic Challenge of NIFTP: Why Frozen Section Fails to Achieve Definitive Identification

The introduction of NIFTP profoundly changed the diagnostic landscape of follicular-patterned thyroid lesions. Defined by strict histopathological criteria—complete encapsulation, absence of capsular or vascular invasion, no true papillae, and uniform follicular growth—NIFTP represents a biologically indolent neoplasm whose diagnosis can only be established after exhaustive sampling of the entire tumor capsule. This fundamental requirement renders intraoperative FS inherently unsuitable for its detection [25].

As demonstrated across multiple studies, FS lacks the ability to reliably evaluate the architectural and capsular features necessary for distinguishing NIFTP from invasive encapsulated follicular variant papillary thyroid carcinoma (EFVPTC). Freezing artifacts can blur delicate nuclear features, distort the capsule, and make it difficult to assess small foci of invasion. It has been emphasized that the mandatory exclusion of papillary structures and the need for complete histologic evaluation cannot be met with FS, making any intraoperative attempt at NIFTP classification technically unsound [26].

Likewise, even extensive intraoperative sampling fails to ensure correct categorization, as the definitive diagnosis hinges on full circumferential capsule examination and a meticulous search for foci of invasion [27].

More recently, Andrade de Almeida et al. (2025) reaffirmed that FS cannot distinguish NIFTP from EFVPTC, and may even mislead surgical decision-making when focal artifacts mimic or obscure invasive features [28].

From a surgical perspective, these limitations have practical implications. Because FS cannot confirm NIFTP—and even more importantly, cannot exclude invasion—its use does not assist intraoperative decisions regarding surgical extent. The modern management of NIFTP favors thyroid lobectomy, and FS cannot provide any information that would appropriately alter this minimally invasive approach. Attempting to diagnose NIFTP intraoperatively both risks overtreatment (conversion to total thyroidectomy based on misleading frozen interpretations) and undermines the accuracy of permanent histology due to tissue distortion [29].

Taken together, current evidence demonstrates that the diagnostic framework of NIFTP is fundamentally incompatible with intraoperative frozen section. Rather than refining the evaluation of indeterminate nodules, FS adds no clinically meaningful information in this context and may, in selected cases, introduce avoidable diagnostic uncertainty. Consequently, the recognition of NIFTP further narrows the already limited indications for FS in contemporary thyroid surgery.

The Table 3 summarizes the studies concerning FS limitations in NIFTP.

### 3.4. Frozen Section for Central Compartment Lymph Nodes in PTC

The application of FS for intraoperative evaluation of central compartment lymph nodes in clinically node-negative papillary thyroid carcinoma (cN0 PTC) was first established by Raffaelli et al., who demonstrated that FS of ipsilateral level VI nodes can accurately identify clinically relevant metastases while sparing patients unnecessary bilateral dissections. In their initial proof-of-concept study, FS showed 80.7% sensitivity and 100% specificity, with false negatives largely confined to micrometastases, a finding of marginal clinical relevance [30]. These results were subsequently confirmed and refined in a prospective series, in which FS again proved reliable in detecting macrometastatic disease and effectively stratifying patients according to the need for unilateral versus bilateral central neck dissection (CND) [31]. In view of the limited reliability of intraoperative nodal assessment [32], these studies established FS as a targeted intraoperative staging tool capable of balancing oncologic adequacy with reduced surgical morbidity.

The clinical implications of this strategy were next expanded to patients with unifocal cN0 T1 PTC scheduled for lobectomy, in whom FS reliably detected macrometastases (>2 mm). This enabled real-time escalation to total thyroidectomy with bilateral CND, thereby reducing the need for staged completion surgery, while most false-negative findings involved only micrometastases of limited clinical impact [33]. Finally, in a propensity-matched case–control study, FS-guided intraoperative decision-making achieved staging accuracy and short-term oncologic outcomes comparable to those obtained with upfront total thyroidectomy and systematic CND, but with lower morbidity. FS was thus positioned as an instrument of de-escalation in low-risk PTC [34].

Beyond the institutional setting in which the FS-guided nodal strategy was originally developed, other groups have explored the feasibility and clinical value of intraoperative central-node frozen analysis in cN0 PTC, providing complementary perspectives.

A protocol from Kim et al. emphasized the meticulous quantification of nodal burden and metastatic focus size. This series demonstrated high sensitivity and specificity with a <5 mm cutoff to define clinically relevant nodal disease. Then, it supported the use of FS to trigger immediate completion thyroidectomy when multiple or sizeable metastases were identified [35].

A more pragmatic and cautious perspective was offered by Yang et al. He found that, although ipsilateral central metastases were relatively common in unilateral low-risk PTMC, FS altered postoperative management in only a small fraction of patients, such as young males or those with lower-pole tumors. These findings reinforce that the incremental therapeutic impact of FS in microcarcinoma is limited and highly context-dependent, even when technically feasible [36]. Taken together, the comparison of these findings with previous evidence demonstrates that the clinical value of frozen section varies markedly across different risk categories, tumor profiles, and institutional settings.

Despite these uncertainties, the high specificity of FS in broader clinical settings was recently confirmed, with false-negative results largely limited to micrometastases, thereby reinforcing the reproducibility of previously established evidence without introducing major conceptual innovation [37].

However, despite these context-related limitations, recent evidence indicates that the FS diagnostic reach may extend further than previously assumed. Peng et al. demonstrated that, beyond central compartment staging, intraoperative assessment of central lymph nodes can also predict Lateral Lymph-Node Metastasis (LLNM). By introducing quantitative cutoffs—including a metastatic focus diameter >2 mm, more than five positive central nodes, and a metastatic ratio >0.53—they showed that FS can stratify the risk of LLNM with good discriminative performance and excellent concordance with final histology (Kappa 0.918). These findings suggest that FS may support selective lateral neck dissection in a subset of higher-risk patients, thereby extending its utility into more complex surgical decision-making scenarios [38].

Finally, recent evidence further expands the potential intraoperative value of FS. It has been shown that FS can also inform multiple prognostic variables, such as the number of involved nodes, extranodal extension, and multifocality, achieving a sensitivity of approximately 80%, a specificity of 100%, and an overall diagnostic accuracy close to 90%. By integrating these parameters into a multiparametric intraoperative model (AUC ~0.85), FS can support tailored surgical decision-making and reduce the likelihood of second-stage surgery in selected patients [13].

The Table 4 summarizes the studies addressed to central lymph node FS assessment. 

### 3.5. Frozen Section for High-Risk Histopathological Features

Despite the long-standing interest in identifying high-risk features intraoperatively, the literature evaluating the ability of frozen section to detect aggressive histopathologic characteristics in thyroid carcinoma is remarkably limited. In a study restricted to follicular-patterned lesions, it has been showed that FS fails to identify capsular or vascular invasion in more than 95% of cases [39].

A study from Park et al. provided one of the few systematic FS evaluations for detecting a high-risk histological feature such as extrathyroidal extension (ETE). In a cohort of 268 PTC, FS identified ETE with 66% sensitivity but 99% specificity, yielding a positive predictive value of 98% and correctly prompting escalation of surgery in real time. False negatives largely reflected subtle or microscopic ETE, which became evident only on permanent sections. These findings show that FS can reliably confirm, though not exclude, clinically relevant ETE and may support intraoperative decision-making in selected patients [40].

In light of the limited data obtained in our research, aggressive tumors of both follicular and Hürthle cell types cannot be identified with sufficient accuracy using FS. With regard to PTCs, any aggressive characteristics can be detected using FS with high specificity; however, the sensitivity was not sufficient to include this investigation among the standards of care.

The main topics examined in the two studies addressed to High-Risk Thyroid cancers are summarized in the Table 5.

### 3.6. Frozen Section in Medullary Thyroid Carcinoma

Medullary thyroid carcinoma (MTC) exhibits early and frequent lymph-node metastasis, making the identification of reliable pre- or intraoperative markers highly desirable to appropriately tailor the extent of surgery. Scheuba et al. [41] demonstrated that the desmoplastic stromal reaction (DSR) can serve as a highly specific intraoperative marker in MTC. In their prospective cohort of 120 patients, all DSR-negative tumors were node-negative (specificity 100%), whereas DSR positivity correlated with a substantially higher risk of lymph-node metastasis. Because DSR reflects a biologically driven fibrotic response mediated by paracrine activation of myofibroblasts (IGF/TGF), its absence may reliably identify a biologically indolent subset of MTC suitable for less extensive neck dissection.

In a subsequent study, the interest of DSR in MTC shifted from a binary assessment to a quantitative model. A 20% DSR threshold was identified, with 96% sensitivity, 60% specificity, and a 94% negative predictive value for lymph-node metastasis. Although this analysis was performed on permanent sections, the authors note that desmoplasia is largely preserved in frozen section, suggesting that this quantitative cutoff may, at least in principle, be translated into intraoperative practice [42].

Additional insights emerge from a recent systematic review, which synthesizes experimental and clinical data to refine the prognostic role of DSR in MTC by integrating clinical, histopathologic, and molecular evidence. Beyond confirming the strong association between DSR and lymph-node metastasis, the review demonstrates that its absence in FS predicts node-negative disease with near-perfect accuracy, supported by >98% concordance between FS and permanent diagnoses. The analysis also validates a quantitative threshold of 10% stromal involvement as a practical discriminator of metastatic potential, with high sensitivity and negative predictive value. Importantly, DSR is contextualized within a broader biologic framework involving fibroblast activation, extracellular-matrix remodeling, and hypoxia-related pathways, thereby elevating it from a purely morphologic observation to a mechanistically grounded surrogate of invasiveness. These insights facilitate a risk-adapted surgical strategy in which DSR-negative tumors may be safely managed with more limited surgery, and they further support the conceptualization of sporadic noninvasive medullary thyroid neoplasm (SNIMTN) as a distinct, biologically low-risk entity [43].

An expanding body of literature consistently demonstrates that DSR is one of the most powerful histopathologic correlates of metastatic potential and prognosis in MTC. Early studies established its strong association with lymph-node involvement, while subsequent quantitative analyses refined threshold values capable of discriminating low- and high-risk tumors. More recent molecular and transcriptomic investigations further reinforce the biological significance of DSR, revealing its link with pathways governing invasion, extracellular-matrix remodeling, and tumor progression. Although most data derive from permanent histology rather than intraoperative assessment, the cumulative evidence underscores DSR as a robust and reproducible marker whose relevance is continuously strengthened across increasingly diverse methodological settings. [44,45,46].

These emerging insights may challenge current clinical guidance, which remains largely reluctant to adopt a conservative surgical approach in medullary thyroid carcinoma [47].

Table 6 summarizes the results of main studies concerning tha application of FS in MTC.

### 3.7. Current Recommendations from International Guidelines

Across major international guidelines, intraoperative frozen section occupies a distinctly marginal position compared with preoperative ultrasound, cytology, and, more recently, molecular testing. The 2015 American Thyroid Association (ATA) guidelines acknowledge that intraoperative evaluation, with or without frozen section, may occasionally confirm malignancy in cytologically indeterminate nodules and allow immediate conversion from lobectomy to total thyroidectomy; however, they do not advocate routine use of FS in this setting [5].

The AAES 2020 guidelines mention FS only within the general framework of intraoperative pathological evaluation, without providing specific recommendations regarding its use to guide surgical extent in PTC [48].

The absence of FS analysis from several contemporary national guidelines appears to show its marginal role in modern thyroid cancer management. Notably, the 2022 Polish National Guidelines—one of the most comprehensive European documents—do not reference frozen section in any diagnostic, surgical, or pathological context. This silence seems to be consistent with the broader trend among international recommendations, in which intraoperative pathology has been progressively de-emphasized in favor of preoperative risk stratification, cytologic classification, and molecular profiling [49].

Consistent with other European guideline statements, the Italian consensus does not attribute a meaningful intraoperative role to frozen section, reflecting the broader view that its diagnostic contribution in thyroid carcinoma is minimal and does not warrant formal recommendation [50].

Recommendations of the Francophone Association of Endocrine Surgery (AFCE) consensus (2023) are broadly aligned: routine FS of the thyroid nodule is not advised in Bethesda II–III and IV nodules, due to its low sensitivity (R.7.6). In the presence of a Bethesda V result, it depends on the context and the extent of surgery previously agreed upon with the patient; however, the result is useful only in case of positivity (R.7.8) [51].

Although FS has largely faded from routine use in the work-up of nodules suspicious for differentiated thyroid carcinoma, its role is being reconsidered in two specific settings: MTC and the intraoperative assessment of Level VI lymph nodes.

Thus, while formal guideline support is absent, the selective use of FS emerges as a center-dependent adjunct reserved for highly experienced teams and for carefully selected early-stage MTC cases [52].

With regard to FS of central compartment (Level VI) lymph nodes, its use remains far from routine practice. Nonetheless, several guidelines have begun to acknowledge it as a legitimate option. Notably, the most recent ATA guidelines on differentiated thyroid cancer implicitly open the door to its selective use. Recommendation 19 states that prophylactic Level VI dissection may be considered in larger or more extensive tumors, “or for whom the information will be used to plan further steps in therapy.” This wording recognizes that, when a central node compartment is removed, additional intraoperative diagnostic assessment, potentially including histological evaluation through frozen section, may be justified to inform subsequent surgical decision-making [53].

Thus, while contemporary guidelines substantially limit the use of FS for nodules suspicious for differentiated thyroid cancer, two scenarios—medullary carcinoma and central compartment (Level VI) node evaluation—seem to signal a modest but noteworthy revival of this intraoperative tool.

The main statements of international guidelines concerning the applications of FS in Thyroid cancer are summarized in the Table 7.

### 3.8. Future Directions and Emerging Roles

As the diagnostic landscape of thyroid disease evolves, emerging technologies are reshaping—not replacing—the intraoperative role of FS. Advances in molecular profiling and quantitative imaging, together with deep-learning systems capable of replicating or exceeding human pattern recognition, now provide preoperative insights that contextualize and enhance intraoperative histology. Recent AI models trained on cytologic and histopathologic images have demonstrated high accuracy in identifying papillary thyroid carcinoma features and predicting malignancy risk, thereby embedding FS within a broader, data-driven framework for surgical planning rather than positioning it as an isolated diagnostic tool [17,18,54]. In this integrated paradigm, intraoperative assessment becomes one component of a multiparametric decision-support strategy increasingly informed by robust preoperative prediction models. Recent work has shown that deep-learning architectures trained on cytologic and histopathologic images can achieve high diagnostic accuracy in distinguishing benign from malignant thyroid lesions. The model proposed by Li et al. integrates multi-scale image features to reproduce key nuclear signatures of papillary thyroid carcinoma features that historically required intraoperative examination. By providing reliable, reproducible preoperative predictions, such systems may further limit the scenarios in which FS offers incremental value [55].

Building on the growing interest in data-integrated intraoperative decision support, recent work has shown that the diagnostic contribution of FS can be substantially amplified when incorporated into multivariable predictive models. In a large cohort of AUS/FLUS nodules, an integrated model combining clinical, ultrasound, and FS features markedly outperformed FS alone (AUC 0.92–0.95), demonstrating that FS, traditionally used as an isolated histologic test, can function as a high-value input within real-time quantitative risk stratification. This framework anticipates a future in which frozen section becomes one component of a multimodal intraoperative ecosystem rather than a stand-alone determinant of surgical extent [56].

A recent study developed the first deep-learning classifier specifically trained on thyroid FS, using 4409 WSIs to categorize lesions into five classes (PTC, MTC, ATC, follicular tumor, and non-cancerous tissue). The model—based on a ResNet101 backbone—achieved high diagnostic performance (accuracy 94.6%, precision 94.7%, AUC 0.996) and preserved accuracy even for papillary microcarcinomas as small as 2 mm, demonstrating clinically relevant sensitivity. Importantly, the entire workflow (tile extraction + inference) was optimized to return a slide-level diagnosis in under 10 min, compatible with intraoperative decision-making. These results suggest that AI-augmented FS interpretation may substantially enhance the speed, consistency, and granularity of intraoperative pathology, supporting its integration into real-time surgical planning [57].

In a significant conceptual extension of the FS-based central compartment assessment pioneered by Raffaelli et al. [30,31,32,33,34], Liu et al. developed a deep-learning model (ThyNet-LNM) that predicts cervical lymph-node metastasis directly from intraoperative frozen sections of the primary PTC tumor, rather than from lymph-node tissue itself. Trained on 1987 whole-slide images and validated across four independent cohorts, the model achieved AUCs of 0.76–0.81, consistently outperforming preoperative ultrasound and CT. Importantly, ThyNet-LNM reduced the rate of unnecessary prophylactic central neck dissections in cN0 T1–T2 PTC from 56.4% to 14.9%, demonstrating its potential as an intraoperative decision tool. From a practical standpoint, this work reinforces, and technologically amplifies, the clinical rationale underlying FS of ipsilateral level VI nodes: both approaches aim to refine intraoperative staging and personalize the extent of surgery. While Raffaelli’s method relies on direct pathological evaluation of nodal tissue, Liu et al. show that AI-enhanced interpretation of tumor-based FS can infer nodal risk by extracting microscopic patterns invisible to the human eye (including tumor–microenvironment interactions). ThyNet-LNM can be viewed as an evolution of the same intraoperative logic, transforming FS from a purely diagnostic tool into a predictive platform capable of guiding selective central neck dissection [58].

Emerging technologies integrating frozen section into multimodal decision-support systems are summarized in Table 8.

## 4. Discussion

Thyroid surgery is generally regarded as a safe and standardized procedure, with low rates of major complications when performed in high-volume centers. Nevertheless, clinically relevant morbidity, including hypoparathyroidism, bleeding and recurrent laryngeal nerve injury, continues to represent a non-negligible concern, particularly as surgical indications expand and treatment paradigms evolve toward greater individualization [59,60,61,62,63,64,65].

In parallel, increasing attention has been directed toward an integrated ontogenetic perspective of thyroid tumorigenesis and precise risk stratification, including the association between thyroid cancer and other malignancies, with the aim of a tailored surgical decision-making [66,67].

The present narrative review synthesizes a heterogeneous but substantial body of evidence addressing the intraoperative role of FS in thyroid surgery across multiple clinical contexts—from indeterminate cytology and NIFTP-related diagnostic challenges to targeted central lymph-node assessment and high-risk histopathological signatures, including MTC.

Several coherent patterns emerge from the present review.

First, FS shows high specificity but persistently limited sensitivity in indeterminate thyroid nodules (Bethesda III–IV) [9,10,12,24,68] and in “suspicious for malignancy” cytology, confirming that its value is essentially confined to confirmatory rather than exclusionary diagnostics. This aligns with historical and contemporary evidence and explains the absence of strong recommendations in international guidelines [48,49,50,51].

Second, FS of the central compartment lymph nodes demonstrates a meaningful intraoperative role: accurately detecting macrometastatic disease, preventing unnecessary bilateral dissections, and reducing the need for staged completion surgery [30,31,32,33,34,35,36,37,38]. These findings define an area in which FS changes operative strategy and stands in contrast to its limited value in follicular-patterned lesions [34,38].

Third, the review highlights the diagnostic impossibility of identifying NIFTP intraoperatively, owing to the requirement for exhaustive capsular evaluation and the subtlety of nuclear features—another domain in which FS cannot compete with definitive histology [25,26,27,28,29].

Fourth, in medullary thyroid carcinoma, FS has no established role in routine surgical planning, though the assessment of desmoplastic stromal reaction (DSR) may hold prognostic significance. However, even in this context, the present review shows that the literature remains sparse and far from guideline integration [41,42,43,44,45,46].

Moreover, emerging technologies—including molecular profiling, radiomics, integrated prediction models, and AI-directed interpretation of FS slides—suggest that the future of intraoperative decision-making will increasingly rely on combined information streams, in which FS contributes as a complementary element rather than a stand-alone determinant. Several recent AI studies demonstrate that FS images can be leveraged to predict malignancy, aggressiveness, and even lymph-node metastasis with high precision, thereby signaling a paradigmatic shift toward multimodal intraoperative decision-support systems [56,57,58,59,60,61,69,70,71,72].

Finally, it should be acknowledged that intraoperative frozen section is currently evolving within a diagnostic landscape that increasingly relies on refined preoperative assessment. In selected settings, thyroid core-needle biopsy (CNB) may provide preoperative histological information, including tissue architecture and the possibility of ancillary analyses such as immunohistochemistry, potentially complementing cytology in indeterminate nodules [73]. However, CNB remains an invasive technique requiring specific expertise and is not routinely applicable to small thyroid nodules or to small-volume lymph-node disease, which represents one of the main targets of intraoperative decision-making in thyroid cancer surgery [73,74]. Therefore, CNB should be regarded as a complementary preoperative option in carefully selected cases rather than an alternative to the intraoperative role historically attributed to frozen section.

This review has several limitations.

Its narrative design precludes formal assessment of bias, meta-analytic synthesis, or strength-of-evidence grading. The included studies demonstrate marked heterogeneity in design (retrospective cohorts, single-center experiences, diagnostic accuracy studies, feasibility trials), patient selection, FS technique, and pathological endpoints. Reporting standards are inconsistent, limiting comparability across cohorts. Many studies rely on institution-specific expertise in FS, reducing generalizability.

Furthermore, the literature on certain key topics—such as FS in high-risk histotypes and in medullary carcinoma—is limited and dated, preventing firm conclusions.

Lastly, rapidly evolving technologies (radiomics, AI, integrated models) introduce conceptual rather than fully validated clinical frameworks, which should be interpreted as promising directions rather than established practice [55,56,57,58].

In conclusion, FS retains a circumscribed but meaningful role in thyroid surgery.

It is unreliable for the intraoperative diagnosis of follicular-patterned lesions, ineffective for identifying NIFTP, and unnecessary when preoperative cytology or molecular testing establishes the diagnosis. However, FS is clinically impactful when applied selectively to central lymph-node assessment in PTC, where it can guide the extent of dissection, reduce overtreatment, and prevent second-stage surgery.

The integration of FS within multimodal predictive frameworks, including molecular markers, radiomics, and AI-augmented image analysis, is reshaping its future role from a stand-alone histologic test to a component of comprehensive intraoperative decision-making.

As guidelines progressively deprioritize routine FS, future research should aim to clarify when its selective use continues to offer unique intraoperative value and how emerging technologies may further refine its interpretation.

## Figures and Tables

**Table 1:** Reappraisal of Frozen Section: Historical Context and Contemporary Criticisms. Historical and contemporary evidence highlighting the progressive reduction of the clinical role of frozen section (FS) in thyroid surgery. Among the included studies, only Zhu et al (2023) [18] attributed a direct positive impact on surgical decision-making to frozen section, although contemporary genetic and biomolecular algorithms currently play a predominant role.

First Author (Year)	Study Type	Clinical Setting	Main Limitation of FS	Impact on Surgery	Key Conclusion
Li Volsi (2005) [15]	Narrative review	Thyroid nodules	Low sensitivity	Minimal	FS rarely changes management
Crowe (2011) [16]	Retrospective	Indeterminate nodules	Structural limitations	None	FS limitations persists despite preoperative risk stratification
Zakka (2023) [17]	evidence-based, practice-oriented recommendations	Indeterminate nodules	Freezing artifacts	Only specific settings	selective, judicious use of FS
Zhu (2023) [18]	Retrospective	Thyroid nodules	Technical artifacts	Yes	FS included in surgical procedure
Mallick (2019) [19]	Retrospective cohort	Thyroid nodules	Diagnostic discordance	Clinically misleading	FS adds little beyond FNA and molecular tests

**Table 2:** Frozen Section in Indeterminate or Suspicious Thyroid Cytology (Bethesda III–IV–V). Diagnostic performance of frozen section in indeterminate and suspicious thyroid cytology, showed high specificity, but limited/low impact on surgery in real scenarios.

First Author (Year)	Bethesda Class	Sample Size	Sensitivity (%)	Specificity (%)	Surgical Impact
Abu-Ghanem (2016) [20]	V	47	65.7	100	Limited
Goemann (2022) [11]	III–V	2040	25	~100	Minimal
Eilsberger (2021) [21]	III–IV	142	>80	100	Negligible
Najah (2019) [10]	III–IV	—	Low	High	None
Hacihasanoglu (2023) [24]	III–IV–V	81	68	95	Limited
Uludag (2023) [12]	III–V–V–VI	__	Low	High	Minimal

**Table 3:** The Diagnostic Challenge of NIFTP: Why Frozen Section Fails. Structural and conceptual incompatibility between frozen section and definitive diagnosis of NIFTP.

First Author (Year)	Study Type	FS Feasibility	Main Limitation	Conclusion
Pusztaszeri (2019) [26]	Review	No	Capsular assessment	FS unsuitable
Rosario (2019) [27]	Review	No	Nuclear criteria	FS incompatible
Andrade de Almeida (2025) [28]	Retrospective Cohort	No	Sampling artifacts	Permanent sections requested
Van Den Berg (2025) [29]	Review	No	Molecular mismatch	FS conceptually obsolete

**Table 4:** Frozen Section for Central Compartment Lymph Nodes in PTC. Evidence supporting frozen section of central compartment lymph nodes as a targeted intraoperative staging tool in selected PTC patients. Highlighted in yellow, Yang et al. is the only study expressing reservations regarding this practice.

First Author (Year)	Study Design	Sensitivity (%)	Specificity (%)	Clinical Role
Raffaelli (2013) [30]	Retrospective	80.7	100	Proof of concept
Raffaelli (2015) [31]	Prospective	65	100	Surgical tailoring
De Crea (2017) [32]	Review	—	—	Concept validation
Raffaelli (2021) [33]	Case–control	80	100	De-escalation
Raffaelli (2021) [34]	Propensity matched	>87%	100	Reduced morbidity
Kim (2022) [35]	Retrospective	>94%	100	Risk-stratified
Yang (2022) [36]	Retrospective	__	__	Strategy Change in 21.6%
Peng (2023) [38]	Quantitative	97.2	96,7	Predictive extension
Jiang (2023) [13]	Retrospective	77.6	100	Multiparametric FS
Pennestrì (2025) [37]	Retrospective Cohort	78	95	Reduced redo-surgery

**Table 5:** Frozen Section for High-Risk Histopathological Features. Scarce evidence supporting the identification of high-risk histopathological features by frozen section.

First Author (Year)	Histotype	Feature Assessed	FS Accuracy	Limitation	Conclusion
Baloch (2002) [39]	FTC/Hürthle	Capsular invasion	Low	Sampling	FS unreliable
Li Volsi (2005) [15]	FTC	Extra-thyroid extension	Very low	Technical	FS inadequate

**Table 6:** Frozen Section in Medullary Thyroid Carcinoma. Desmoplastic stromal reaction as a prognostic marker in medullary thyroid carcinoma and its potential intraoperative relevance. LN Mts = Lymph Node Metastasis. I.O. Prognostic Marker = IntraOperative Prognostic Marker.

First Author (Year)	Marker	FS Applicability	Sensitivity (%)	Prognostic Value	Conclusion
Scheuba (2006) [41]	DSR	Yes	Low	High	Roule out marker
Aubert (2018) [42]	DSR	Indirect	Not applicable	Strong	Correlates with LNM
Machens (2024) [43]	DSR	Yes	100 (LN Mts)	Very high	I.O. Prognostic marker
Procopio (2025) [44]	DSR	Indirect	100	Very high	Tailored dissection
Niederle (2025) [46]	DSR	Yes	Yes	Strong	Tailored Surgery
Machens (2025) [45]	DSR	Yes	Not applicable	High	Intraoperative management

**Table 7:** International Guidelines and Consensus Statements on Frozen Section. Frozen section is largely absent or marginally addressed in contemporary international guidelines I.O.: Intraoperative; Central L.N.: Central Lymph Nodes.

Guideline	Year	FS Mention	Recommendation	Tone
ATA	2015	Explicit (nodes)	Not systematic	Discouraging
AAES	2020	Within I.O. evaluation	Limited	Neutral
Polish consensus	2022	None	—	Silent
Italian consensus	2023	None	—	Silent
French consensus	2023	Explicit (nodes)	Only in Bethesda V	Moderately Favorable
KAEK, BAETS, etc.	2022	In MTC	Emerging role	Emerging role
ATA	2025	Implicit	Central L.N.	Supportive

**Table 8:** Future Directions and Emerging Roles Integrating Frozen Section. Emerging technologies reframing frozen section as part of integrated intraoperative decision-support systems. D.L./WSI = Deep Learning/Whole Slide Images; LNM: Lymph Node Metastasis; LND: Lymph Node Dissection; I.O. = intraoperative; NPV = Negative Predictive Value.

First Author (Year)	Technology	Input Data	Outcome	Performance	Relationship to FS and Utility
Sorrenti (2022) [57]	AI/radiomics	US	LNM	AUC = 0.87	Early risk stratification
He (2024) [59]	D.L./WSI	WSI-FS	Assisted stratification	AUC = 0.995	Improved sensitivity
Li (2024) [58]	Predictive model	TG/US/FS	I.O. rule out FTC	NPV = 94.1%	FS: contextualized and strengthened
Li (2025) [60]	Statistical learning model AI-based	Clinical, Molecular, US, FS	Risk stratification	PPV = 99% NPV = 74%	Enhances FS utility
Liu (2023) [61]	D.L./WSI	Multicentric dateset FS	LNM	AUC = 0.76–0.81	Unnecessary LND from 56 to 15%

## Data Availability

The original contributions presented in the study are included in the article, further inquiries can be directed to the corresponding authors.
